# Myocardial infarction following COVID-19 vaccine administration; a systematic review

**DOI:** 10.1016/j.heliyon.2022.e11385

**Published:** 2022-11-11

**Authors:** Dana H. Baqi, Fahmi H. Kakamad, Zana H. Mahmood, Fattah H. Fattah, Shaho F. Ahmed, Marwan N. Hassan, Bnar J. Hama Amin, Shvan H. Mohammed, Tomas M. Mikael, Hunar A. Hassan, Abdulwahid M. Salh

**Affiliations:** a Smart Health Tower, Madam Mitterrand Street, Sulaimani, Kurdistan, Iraq; b College of Medicine, University of Sulaimani, Sulaimani, Kurdistan, Iraq; c Kscien Organization, Hamdi Str., Azadi Mall, Sulaimani, Kurdistan, Iraq; d Molecular Diagnostic Laboratory, Sulaimani Veterinary Directorate, Sulaimani, Kurdistan, Iraq

**Keywords:** COVID-19, SARS-CoV-2, Cardiac, Myocardial infarction, Vaccination

## Abstract

**Introduction:**

Covid-19 vaccines have been assessed in randomized trials, which are designed to establish efficacy and safety, but are insufficient in power to detect rare adverse outcomes. Among the adverse cardiac events associated with mRNA COVID-19 vaccines are inflammations (e.g., pericarditis or myocarditis), thrombosis, and ischemia.

**Objective:**

This systematic review aims to evaluate the reported cases of myocardial infarction (MI) after COVID-19 vaccinations.

**Method:**

Web of Science, MEDLINE on OVID, PubMed, and Google Scholar were searched for English-language papers published until March 25, 2022.

**Results:**

This study included 15 papers (10 case reports and 5 case series). In total, 20 individuals were included who had received COVID-19 vaccines and experienced MI. Males (55%) reported more adverse occurrences than females (45%) across the majority of event categories. The mean time from the administration of the vaccine to the onset of symptoms was 2 days (0–10 days). The AstraZeneca vaccine was responsible for more than half of the reported events. In the majority of cases, the event developed after receiving the first dose of vaccination.

**Conclusion:**

MI related to COVID19 vaccination is a rare, but serious and life-threatening condition. Chest discomfort should be regarded as a warning sign, particularly in people who have been administered a dose of the vaccine within the previous two days.

## 1 Introduction

Coronavirus disease 2019 (COVID-19), a SARS-CoV-2 RNA virus-associated acute respiratory disease, emerged in December 2019 in Wuhan, China, and spread rapidly throughout the world. Since its inception, as of April 1, 2022, it has infected more than 486 million people and caused around 6.1 million deaths [1]. The high incidence of COVID-19 promotes the need for vaccination as soon as possible to combat the COVID-19 pandemic. At the moment, vaccination against SARS-CoV-2 that induces an immune response against the SARS-CoV-2 spike protein is the principal approach to combating the COVID-19 pandemic. Real-world trials have found that vaccination is effective in preventing COVID-19 [2]. Currently, over thirteen COVID-19 vaccines have been approved for emergency use in various countries, including Comirnaty (BNT162b2), Moderna COVID-19 Vaccine (mRNA-1273), COVID-19 Vaccine AstraZeneca (ChAdOx1 nCov-19), also known as Covishield, and Sputnik V [3]. Covid-19 vaccines have been assessed in randomized trials designed to establish efficacy and safety but are insufficient in power to detect rare adverse outcomes [4]. The majority of reported adverse events were minimal local or systemic reactions, such as injection site discomfort, erythema, edema, fever, headache, and myalgia. However, a wide range of adverse events and consequences have been documented for COVID-19 vaccines, including acute myocardial infarction (MI), pulmonary embolism, stroke, and venous thromboembolism [5]. Despite the overall effectiveness of vaccinations, investigating the safety of COVID-19 vaccinations has shown significant cardiac adverse effects [6]. Inflammations (e.g., pericarditis or myocarditis) thrombosis, and ischemia are among the adverse cardiac events related to mRNA COVID-19 vaccinations [5].

This review aims to assess the reported cases of MI following the administration of the COVID-19 vaccines.

## 2 Method

### 2.1 Study design

The current systematic review followed the Preferred Reporting Items for Systematic Reviews and Meta-Analyses (PRISMA) guidelines [7].

### 2.2 Literature search

Up to March 25, 2022, the PubMed, Scopus, and Web of Science databases with Google Scholar were searched for papers describing MI following COVID-19 vaccination. The search terms were COVID-19, SARS-CoV-2, nCOV-19, vaccine(s), vaccination(s), cardiac, cardiovascular, myocardial injury, myocardial infarction, ischemia, Pfizer, BNT162b2, Moderna, mRNA-1273, AstraZeneca, ChAdOx1 nCov-19, long, consequences, sequela, sequelae, adverse effect(s), event(s). References cited in the included studies were used to complement the data collection.

### 2.3 Eligibility

The inclusion criteria for this study focused on case report and case series studies that described MI associated with one or two doses of any COVID-19 vaccine. MI should be recorded within 42 days following vaccination, regardless of age. Excluded studies are those taken from databases containing vaccine-related adverse event reports, as well as studies that were not peer-reviewed and were published in predatory journals.

### 2.4 Study selection

All studies have been found using electronic and manual searches, and all duplicates have been deleted. Two of the authors independently assessed the titles and abstracts of the publications and excluded those that were irrelevant. Following the first screening, the same two authors assessed the full texts of the remaining publications based on the inclusion and exclusion criteria.

### 2.5 Data item

Microsoft Office Excel was used to extract the data. Each study yielded the following information, which were gathered by two authors: (1) basic information about the articles, such as title, first author's name, publication date, study design, and country; (2) characteristics of the presented cases, such as the number of reported cases, age, gender, past medical history, smoking history, name of vaccine received, the dose of vaccine that subsequently manifested symptoms, and time from vaccination to symptom onset; (3) presenting symptoms, such as chest pain; (4) cardiac testing, including electrocardiogram (ECG) and troponin. Other authors rechecked all the extracted data.

## 3 Results

**Figure 1:**
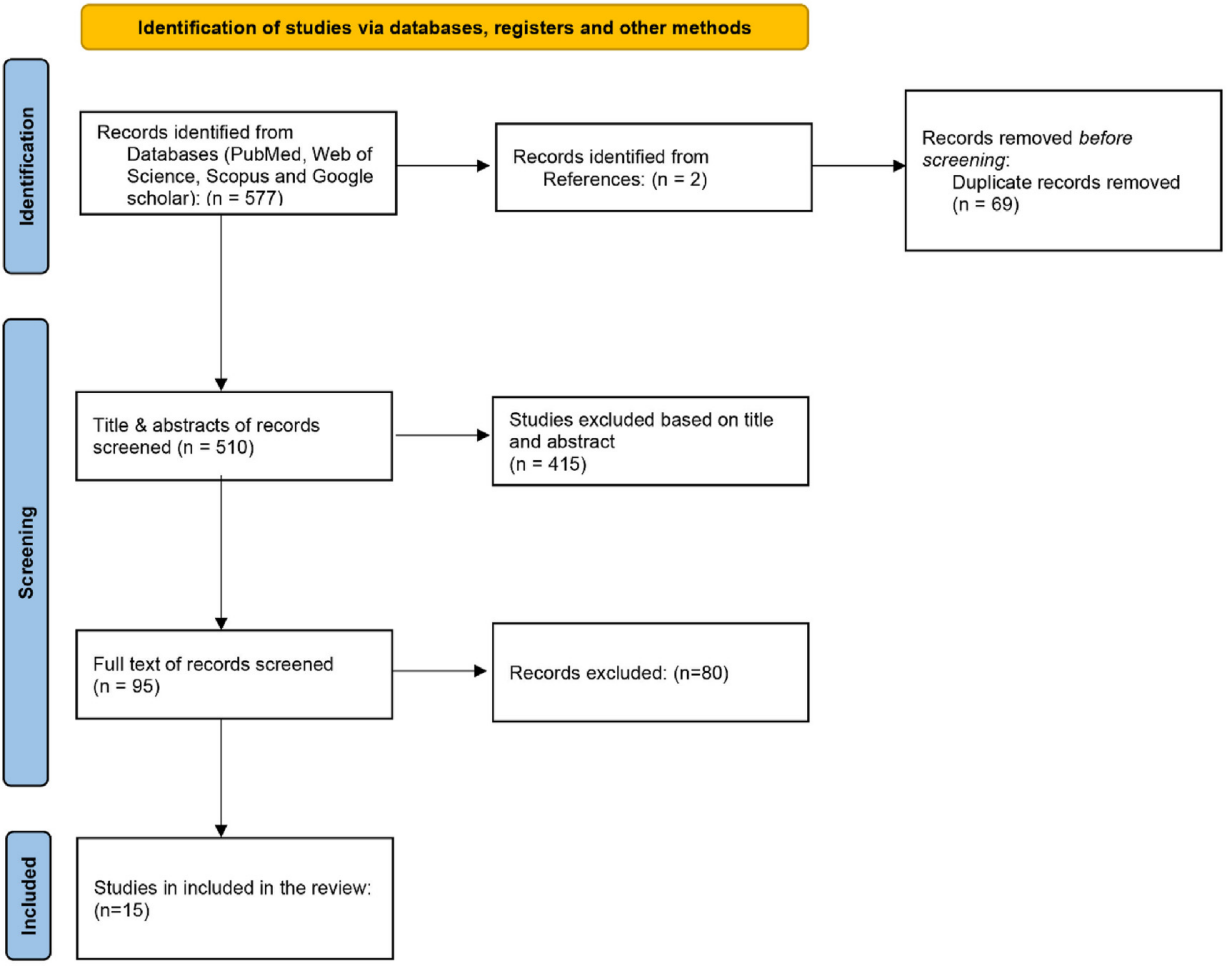
Prisma chart.

During the initial database searches, 577 articles were found (+2 articles from references). The data was gathered from PubMed, Scopus, and Web of Science databases with Google Scholar, and several duplicated papers were included. After deleting 69 duplicates, there were 510 non-duplicate articles. Following a review of the titles and abstracts, 415 irrelevant studies were eliminated from consideration. During the full-text screening process, 80 irrelevant papers were also eliminated. In the final round, 15 papers were included for additional assessment. Figure 1 illustrates the PRISMA chart in further details.

Of the 15 studies included, 5 were case series and 10 were case reports. All were peer-reviewed and were published in 2021–22. Four studies were conducted in India, two in the United States, two in Taiwan, two in the United Kingdom, and five in other countries. The characteristics of the included studies are shown in Table 1.

**Table 1:** The characteristics of included studies.

No	Author	Type of study	Date of publishing	Country	No. of Cases	No. Of cases with MI	Age Mean (year)	Sex (Male)	Duration from vaccination to onset (day)
1	Sung et al [8]	Case series	12-July-21	USA	2	2	55 (42,68)	1	0
2	Kumar et al [9]	Case series	6-Jun-21	India	2	2	68 (60,76)	2	0
3	Lee et al [10]	Case series	30-Jun-21	Singapore	3	2	71.5 (73–70)	0	0
4	Scully et al [11]	Case series	16-Apr-21	UK	23	1	54	1	10
5	Srinivasan et al [12]	Case series	7-Jun-21	India	3	3	56.3 (46-48-75)	2	6
6	Boivin et al [13]	Case report	2-Mar-21	USA	1	1	96	0	0
7	Chatterjee et al [14]	Case report	14 -Apr-21	India	1	1	63	1	2
8	Chiang et al [15]	Case report	30-Sep-21	Taiwan	1	1	75	0	8
9	Flower et al [16]	Case report	27-Sep-21	UK	1	1	40	1	8
10	Hsu et al [17]	Case report	1-Feb-22	Taiwan	1	1	33	1	9
11	Iqbal et al [18]	Case report	24-Jan-22	Pakistan	1	1	61	1	0
12	Maadarani et al [19]	Case report	11-Aug-21	Kuwait	1	1	62	0	0
13	Park et al [20]	Case report	30-Nov-21	Korea	1	1	69	0	0
14	Mungmunpuntipantip et al [21]	Case report	10-Nov-21	India	1	1	72	0	0
15	Tajstra et al [22]	Case report	3-May-21	Poland	1	1	86	1	0

A total of 20 patients were administered COVID-19 vaccines and experienced MI. This problem occurred following vaccination with the BNT162b2, mRNA1273, AstraZeneca ChAdOx1, and Janssen (Ad26COVS1) COVID-19 vaccines. The mean age of the 20 patients with MI was 63.45 years (33–96 years), with the majority being male (55%). The most frequent presenting symptom was chest pain (15, 75%). The average time from vaccination to the onset of symptoms was two days (ranging from 0 to 10 days). AstraZeneca vaccine was responsible for more than half of the reported events (12, 60%), followed by Moderna (4, 20%), and Pfizer (3, 15%). The incidence occurred in 17 individuals following the first dose of vaccination. Overall, 4 (20%) patients were smokers. Three patients had a negative past medical history. Hypertension was the most common comorbid condition (8, 40%), followed by diabetes mellites (8, 40%) and hyperlipidemia (7, 35%). ST-segment elevation in the inferior leads was the most common ECG finding (10, 50%). Troponin was elevated in 11 (55%) cases, normal in one case, and inapplicable in the remainders. In Table 2, the characteristics of patients with MI are shown.

**Table 2:** Demographic features and clinical characteristics of MI patients.

Variables	Number (20)
Age	63.5 (33–96)
Gender	
Male	11 (55%)
Female	9 (45%)
Days from vaccination to symptom onset (days)	2 (0–10)
Country of study	
USA	2 (13%)
UK	2 (13%)
India	4 (27%)
Taiwan	2 (13%)
Other	5 (33%)
Vaccination profile	
AstraZeneca	12 (60%)
Pfizer	3 (15%)
Moderna	4 (20%)
Janssen	1 (5%)
Smoker	
No	12 (60%)
Yes	4 (20%)
N/A	4 (20%)
Past medical history	
Negative	3 (15%)
Hypertension	8 (40%)
Diabetes Mellites	8 (40%)
Hyperlipidemia	7 (35%)
ACS	3 (15%)
Thyroid disease	1 (5%)
12-lead electrocardiogram	
ST segment elevation in	
Inferior leads	10 (50%)
Precordial leads	2 (10%)
ST segment depression precordial leads	1 (5%)
T wave inversion in inferior leads	1 (5%)
Normal	4 (20%)
N/A	2 (10%)
Troponin	
Elevated	11 (55%)
Normal	1 (5%)
N/A	8 (40%)
Vaccine dose	
1^st^ dose	17 (85%)
2^nd^ dose	2 (10%)
N/A	1 (5%)
Presentation	
Chest pain	15 (75%)
Shortness of breath	2 (10%)
Shoulder pain	3 (15%)
Treatment	
Intervention	7 (35%)
Medication	9 (45%)
N/A	4 (20%)
Outcome	
Discharged	10 (50%)
Death	3 (15%)
N/A	7 (35%)

## 4 Discussion

In this systematic review, we discovered that MI following the administration of COVID-19 vaccination occurred most frequently in males with a mean age of 63.45 years. It was most frequently observed after AstraZeneca vaccination. In four cases, ECG was normal, and in one case, troponin was normal. The majority of the events occurred after the first dose of vaccination. The most common presenting indication of the event was chest pain. Patients who had a coexisting disease were more prone to have the complication. More than half of the cases recovered well and were discharged from the hospitals; three of the cases died.

The COVID-19 outbreak has emerged as the most serious source of concern for the world's health and political institutions. The COVID-19 vaccination program was launched in less than a year, marking a significant milestone in research and development. The initial vaccination experience demonstrated that this vaccine was quite safe and well-tolerated [12]. Even though the speed with which vaccines are being developed should be praised, most vaccines have only been approved for emergency use, based on limited trial results [23]. Vaccine safety, like that of other pharmaceutical drugs, must be studied after regulatory approval to support what was established during clinical studies [23]. The early authorization and subsequent global distribution of the SARS-CoV-2 vaccine have resulted in reports of adverse reactions following vaccination. The majority of adverse reactions recorded so far have been associated with risks that are comparable to baseline risks in the general population and have not raised concerns. However, background risks may be difficult to assess or interpret for extremely infrequent events [11]. Although the side effects of these vaccinations are typically mild and temporary, there has been an increase in reports of hemorrhage, thrombotic events, and thrombocytopenia after receiving COVID-19 vaccinations which have prompted some concerns [14].

Although uncommon, vaccine-related thrombo-embolic consequences have been observed with the ChAdOx1 nCoV19 and Ad26.COV2.S vaccines, mostly in Europe and North America [11, 24, 25]. Scully et al. described 23 patients (median age, 46 years) who experienced thrombosis and thrombocytopenia 6–24 days after the first dose of the ChAdOx1 nCoV-19 vaccination. The majority of those who had venous thrombosis (60%) were females. They also mentioned PF-4-dependent ITP as a possible cause of thrombotic events [11].

Other rare side effects have recently been documented, including cerebral venous sinus thrombosis, acute ST-segment elevation MI (STEMI) with massive thrombus in the coronary arteries, and (autoimmune) myocarditis [22, 26]. Several cases of pericarditis and myocarditis have been reported in the United States vaccine adverse effect reporting system (VAERS) as well as in the European database (EudraVigilance) [27]. COVID-19 vaccination may cause an excessive immune reaction in certain individuals, resulting in autoimmune cardiac injury, which is likely to represent autoimmune myocarditis [27]. However, it is still unknown if there is an association between COVID-19 vaccinations and myocarditis. Furthermore, researchers do not know whether various vaccinations are related to distinct immunological pathways that may provoke autoimmune myocarditis and may potentially define the frequency and severity of such an adverse event [27].

There are insufficient scientific data to demonstrate a definitive causal association between COVID-19 vaccination and MI. The vaccination might be a contributory factor by increasing pressure on the heart, but it is not a direct cause of MI [24]. MI following COVID-19 vaccination is not a frequently reported association. Boivin et al. documented a case of a 96-year-old woman in the United States who had an MI 1 h after receiving her first dose of Moderna COVID-19 vaccination [13]. Chatterjee et al. reported another case of a 63-year-old man with no known risk factors for coronary artery disease who developed an acute ST-elevated inferior wall MI two days after receiving the COVID-19 vaccine, Covishield [14]. Early detection of this acute cardiovascular disease may be difficult since injection site discomfort may cause ischemic symptoms, resulting in delayed presentations [8].

Many hypotheses have been proposed to explain the mechanism of MI after COVID-19 vaccination. Some believe that the prothrombotic condition following vaccination is caused by an autoimmune reaction against platelets, which is clinically comparable to autoimmune heparin-induced thrombocytopenia [24, 28]. On the other hand, Boivin et al. hypothesized that stress caused by the COVID-19 vaccine may have resulted in demand ischemia, resulting in cardiac events [14]. Furthermore, MI following COVID-19 vaccination may be due to allergic vasospasm in reaction to the vaccine, known as Kounis syndrome. It has been proposed that high levels of immunoglobulin E antibodies may be a risk factor for MI [29].

Individuals with several co-morbid risk factors are at a higher risk of experiencing an acute MI. The psychological stress of receiving the COVID-19 vaccine can disrupt a chronic stable plaque and turn it into a susceptible plaque. Acute coronary syndrome may be caused by the precipitation of acute inflammation as an immunologic response to the vaccine injection [12].

Mahase et al. stated that it would be inappropriate to suggest a causal association between the COVID-19 vaccination and MI at this time. Given that vaccines are being widely distributed and MI is a relatively common emergency, it might just be a coincidence or perhaps an unusual reaction. As a result, before speaking with the patient's attendant or the general public, each case should be properly evaluated. As a result, these findings should never impede vaccination distribution, but producers and independent authorities should continue to monitor developing evidence before reaching a final determination [30]. Maadarani et al. reported a case of STEMI 1.5 h after AZD1222 COVID-19 vaccination and concluded that it is probably too early to declare that the vaccine is the cause of MI in these few cases described in the literature. Given that MI is a common diagnosis in everyday practice and that mass vaccination against COVID-19 has been administered, a coincidence might be an explanation [19].

In conclusion, although MI related to COVID-19 vaccination is rare, it can be life-threatening. Chest discomfort should be regarded as a warning sign, particularly in people with comorbid conditions and those who have been administered a dose of the vaccine within the previous two days.

## Declarations

### Author contribution statement

All authors listed have significantly contributed to the development and the writing of this article.

### Funding statement

This research did not receive any specific grant from funding agencies in the public, commercial, or not-for-profit sectors.

### Data availability statement

Data included in article/supp. material/referenced in article.

### Declaration of interest’s statement

The authors declare no conflict of interest.

### Additional information

No additional information is available for this paper.

## Appendix A Supplementary data

CAREchecklistEnglish CAREchecklistEnglish

JBI checklist for case series JBI checklist for case series

Naranjo algorythm Naranjo algorythm

who-umc-causality-assessment who-umc-causality-assessment
