# Supplementary material for: Myocardial infarction following COVID-19 vaccine administration; a systematic review
Source: Heliyon. 2022 Nov 11;8(11):e11385. doi: 10.1016/j.heliyon.2022.e11385 (PMC9650518; doi:10.1016/j.heliyon.2022.e11385)
Supplement: JBI checklist for case series [file mmc2.docx]

**Table 1;** JBI checklist questions (Case Series)

| **JBI checklist questions (Case Series)** | **Sung et al** | **Kumar et al** | **Lee et al** | **Scully et al** | **Srinivasan et al** |
| --- | --- | --- | --- | --- | --- |
| 1. Were there clear criteria for inclusion in the case series? | Yes | Yes | Yes | Yes | Yes |
| 1. Was the condition measured in a standard, reliable way for all participants included in the case series? | Yes | Yes | Yes | Yes | Yes |
| 1. Were valid methods used for identification of the condition for all participants included in the case series? | N/A | N/A | Yes | N/A | N/A |
| 1. Did the case series have consecutive inclusion of participants? | N/A | N/A | N/A | Yes | N/A |
| 1. Did the case series have complete inclusion of participants? | Yes | Yes | Yes | Yes | Yes |
| 1. Was there clear reporting of the demographics of the participants in the study? | Yes | Yes | Yes | Yes | Yes |
| 1. Was there clear reporting of clinical information of the participants? | Yes | Yes | Yes | No | Yes |
| 1. Were the outcomes or follow up results of cases clearly reported? | Yes | Yes | Yes | No | Yes |
| 1. Was there clear reporting of the presenting site(s)/clinic(s) demographic information? | Yes | Yes | Yes | No | Yes |
| 1. Was statistical analysis appropriate? | N/A | N/A | N/A | Yes | N/A |
