# Supplementary material for: Myocardial infarction following COVID-19 vaccine administration; a systematic review
Source: Heliyon. 2022 Nov 11;8(11):e11385. doi: 10.1016/j.heliyon.2022.e11385 (PMC9650518; doi:10.1016/j.heliyon.2022.e11385)
Supplement: Naranjo algorythm [file mmc3.pdf]

NARANJO ADVERSE DRUG REACTION PROBABILITY SCALE

The Naranjo ADR Probability Scale was developed to help standardize assessment of causality for all adverse drug reactions. The scale was also designed for use in controlled trials and registration studies of new medications, rather than in routine clinical practice.

Research authors: *Naranjo CA, Busto U, Sellers EM, Sandor P, Ruiz I, Roberts EA, Janecek E, Domecq C, Greenblatt DJ*

RESULT

2022-09-15 13:24

Score 3

Based on the following parameters:

1. Are there previous conclusive reports on this reaction?	Yes +1
2. Did adverse event appear after the suspected drug was given?	Yes +2
3. Did the adverse reaction improve when the drug was discontinued or a specific antagonist was given?	Not known or not done
4. Did the adverse reaction appear when the drug was readministered?	Not known or not done
5. Are there alternative causes that could have caused the reaction?	Yes -1
6. Did the reaction reappear when a placebo was given?	Not known or not done
7. Was the drug detected in any body fluid in toxic concentrations?	Not known or not done
8. Was the reaction more severe when the dose was increased, or less severe when the dose was decreased?	Not known or not done
9. Did the patient have a similar reaction to the same or similar drugs in any previous exposure?	Not known or not done
10. Was the adverse event confirmed by any objective evidence?	Yes +1

Disclaimer: This model is provided for educational, training and information purposes. It must not be used to support medical decision making, or to provide medical or diagnostic services.

OUTCOME STRATIFICATION

Result interval 2 to 4

POSSIBLE ADR

RESULT INTERPRETATION

- ***Doubtful*** *ADR (<2): The reaction was likely related to factors other than a drug).*
- ***Possible*** *ADR (2 to 4): The reaction followed a temporal sequence after a drug, possibly followed a recognized pattern to the suspected drug and could be explained by characteristics of the patient's disease.*
- ***Probable*** *ADR (5 to 8): The reaction followed a reasonable temporal sequence after a drug, followed a recognized response to the suspected drug, was confirmed by withdrawal but not by exposure to the drug, and could not be reasonably explained by the known characteristics of the patient's clinical state.*
- ***Definite*** *ADR (≥9): The reaction followed a reasonable temporal sequence after a drug or in which a toxic drug level had been established in body fluids or tissues, followed a recognized response to the suspected drug and was confirmed by improvement on withdrawing the drug and reappeared on reexposure.*

This model is provided for educational, training and information purposes. It must not be used to support medical decision making, or to provide medical or diagnostic services. Read our full disclaimer at: https://www.evidencio.com/disclaimer.
