# Supplementary material for: Myocardial infarction following COVID-19 vaccine administration; a systematic review
Source: Heliyon. 2022 Nov 11;8(11):e11385. doi: 10.1016/j.heliyon.2022.e11385 (PMC9650518; doi:10.1016/j.heliyon.2022.e11385)
Supplement: who-umc-causality-assessment [file mmc4.pdf]

The use of the WHO-UMC system for standardised case causality assessment

Why causality assessment?

An inherent problem in pharmacovigilance is that most case reports concern *suspected* adverse drug reactions. Adverse reactions are rarely specific for the drug, diagnostic tests are usually absent and a rechallenge is rarely ethically justified. In practice few adverse reactions are 'certain' or 'unlikely'; most are somewhere in between these extremes, i.e. 'possible' or 'probable'. In an attempt to solve this problem many systems have been developed for a structured and harmonised assessment of causality (1). None of these systems, however, have been shown to produce a precise and reliable quantitative estimation of relationship likelihood. Nevertheless, causality assessment has become a common routine procedure in pharmacovigilance. The advances and limitations of causality assessment are reviewed in *Table 1*[(2)].

Table 1. Advances and limitations of standardised case causality assessment

What causality assessment can do	What causality assessment cannot do
Decrease disagreement between assessors	Give accurate quantitative measurement of relationship likelihood
Classify relationship likelihood	Distinguish valid from invalid cases
Mark individual case reports	Prove the connection between drug and event
Improvement of scientific evaluation; educational	Quantify the contribution of a drug to the development of an adverse event
	Change uncertainty into certainty

The WHO-UMC causality assessment system

The WHO-UMC system has been developed in consultation with the National Centres participating in the Programme for International Drug Monitoring and is meant as a practical tool for the assessment of case reports. It is basically a combined assessment taking into account the clinical-pharmacological aspects of the case history and the quality of the documentation of the observation. Since pharmacovigilance is particularly concerned with the detection of unknown and unexpected adverse reactions, other criteria such as previous knowledge and statistical chance play a less prominent role in the system. It is recognised that the semantics of the definitions are critical and that individual judgements may therefore differ. There are other algorithms that are either very complex or too specific for general use. This method gives guidance to the general arguments which should be used to select one category over another.

The various causality categories are listed in Table 2. The assessment criteria of the various categories are shown in a point-wise way, as has been developed for practical training during the UMC Training courses.

Table 2. WHO-UMC Causality Categories

Causality term	Assessment criteria*
**Certain**	• Event or laboratory test abnormality, with plausible time relationship to drug intake<br>• Cannot be explained by disease or other drugs<br>• Response to withdrawal plausible (pharmacologically, pathologically)<br>• Event definitive pharmacologically or phenomenologically (i.e. an objective and specific medical disorder or a recognised pharmacological phenomenon)<br>• Rechallenge satisfactory, if necessary
**Probable/ Likely**	• Event or laboratory test abnormality, with reasonable time relationship to drug intake<br>• Unlikely to be attributed to disease or other drugs<br>• Response to withdrawal clinically reasonable<br>• Rechallenge not required
**Possible**	• Event or laboratory test abnormality, with reasonable time relationship to drug intake<br>• Could also be explained by disease or other drugs<br>• Information on drug withdrawal may be lacking or unclear
**Unlikely**	• Event or laboratory test abnormality, with a time to drug intake that makes a relationship improbable (but not impossible)<br>• Disease or other drugs provide plausible explanations
**Conditional/ Unclassified**	• Event or laboratory test abnormality<br>• More data for proper assessment needed, or<br>• Additional data under examination
**Unassessable/ Unclassifiable**	• Report suggesting an adverse reaction<br>• Cannot be judged because information is insufficient or contradictory<br>• Data cannot be supplemented or verified

*All points should be reasonably complied with

The use of the WHO-UMC system

To illustrate how the system works, we suggest to first making a comparison of the criteria and wording of 'Probable' and Certain'. First of all there is one more criterion in the category 'Certain', the fourth: 'Event definitive pharmacologically or phenomenologically', i.e. an objective and specific medical disorder or a recognised pharmacological phenomenon (for instance 'grey baby syndrome' and chloramphenicol, or anaphylaxis immediately after the administration of a drug that had been given previously). This means that any other event is automatically excluded and can never qualify for 'Certain' (even in the case of a positive rechallenge observation). For 'Certain', rechallenge information with a satisfactory outcome is requested (i.e. what has happened when the drug was first stopped

and later on resumed), unless the evidence in the report is already convincing without a re-exposure.

For 'Probable', on the other hand, a rechallenge is not required. To qualify as 'Certain' the interval between the start of the drug and the onset of the event must be 'plausible'; this means that there is in sufficient detail a positive argument in support of the view that the drug is causally involved, pharmacologically or pathologically. For 'Probable' the time relationship should be 'reasonable'; this is a more neutral term covering everything that is not unreasonable. Also, with regard to the second criterion, 'alternative causes', the wording is different in 'Probable'. For 'Certain' the occurrence of the event cannot be explained by any disease the patient is known to have or any other drug taken. For 'Probable', on the other hand, the event is 'unlikely' to be attributable to another cause. Also the dechallenge situations (i.e. what happened after stopping) are different. In a 'Certain' case report, the course of events constitutes a positive argument in favour of holding the suspected drug responsible, in pharmacological or pathological respects, whereas in a 'Probable' case it is sufficient if it is 'clinically reasonable' (i.e. not unreasonable).

The essential distinctions between 'Probable' and 'Possible' are that in the latter case there may be another equally likely explanation for the event and/or there is no information or uncertainty with regard to what has happened after stopping.

The criteria that may render the connection 'Unlikely' are firstly the time relationship is improbable (with the knowledge at the time), and/or another explanation is more likely. The term 'Unclassified/Conditional' is of a preliminary nature and is appropriate when, for a proper assessment, there is more data needed and such data are being sought, or are already under examination. Finally, when the information in a report is incomplete or contradictory and cannot be complemented or verified, the verdict is 'Unclassifiable'.

Since by far the most frequent categories in case reports are 'Possible' and 'Probable', the usual approach to using the system is to choose one of these categories (depending on the impression of the assessor) and to test if the various criteria fit with the content of the case report. If the report seems stronger one can go one step 'higher' (e.g. from 'Possible' to 'Probable'), if the evidence seems weaker one should try a 'lower' category. To see if that category is the right one or if it does again not seem to fit, the next adjacent term is tried.

For drug-drug interactions the WHO-UMC system can be used by assessing the actor drug, which influences the kinetics or dynamics of the other drug (which has usually been taken over a longer period), in the medical context of the patient.

How does it work?

How the WHO-UMC causality assessment system can be used will be illustrated with the aid of a few real-life case reports. These will be made available on the UMC website in the near future.

1. Meyboom RHB, Royer RJ. Causality Classification in Pharmacovigilance Centres in the European Community. Pharmacoepidemiology and Drug Safety 1992; 1:87-97.
2. Meyboom RHB. Causal or Casual? The Role of Causality Assessment in Pharmacovigilance. Drug Safety 17(6): 374-389, 1997.
3. Edwards IR, Biriell C. Harmonisation in Pharmacovigilance. Drug Safety 10(2): 93-102, 1994.

Summary description of Causality Assessment

Term	Description	Comment
**Certain**	A clinical event, including laboratory test abnormality, occurring in a plausible time relationship to drug administration, and which cannot be explained by concurrent disease or other drugs or chemicals. The response to withdrawal of the drug (dechallenge) should be clinically plausible. The event must be definitive pharmacologically or phenomenologically, using a satisfactory rechallenge procedure if necessary.	It is recognized that this stringent definition will lead to very few reports meeting the criteria, but this is useful because of the special value of such reports. It is considered that time relationships between drug administration and the onset and course of the adverse event are important in causality analysis. So also is the consideration of confounding features, but due weight must be placed on the known pharmacological and other characteristics of the drug product being considered. Sometimes the clinical phenomena described will also be sufficiently specific to allow a confident causality assessment in the absence of confounding features and with appropriate time relationships, e.g. penicillin anaphylaxis.
**Probable/ Likely**	A clinical event, including laboratory test abnormality, with a reasonable time sequence to administration of the drug, unlikely to be attributed to concurrent disease or other drugs or chemicals, and which follows a clinically reasonable response on withdrawal (dechallenge). Rechallenge information is not required to fulfil this definition.	This definition has less stringent wording than for "certain" and does not necessitate prior knowledge of drug characteristics or clinical adverse reaction phenomena. As stated no rechallenge information is needed, but confounding drug administration underlying disease must be absent.
**Possible**	A clinical event, including laboratory test abnormality, with a reasonable time sequence to administration of the drug, but which could also be explained by concurrent disease or other drugs or chemicals. Information on drug withdrawal may be lacking or unclear.	This is the definition to be used when drug causality is one of other possible causes for the described clinical event.

**Unlikely**	A clinical event, including laboratory test abnormality, with a temporal relationship to drug administration which makes a causal relationship improbable, and in which other drugs, chemicals or underlying disease provide plausible explanations.	This definition is intended to be used when the exclusion of drug causality of a clinical event seems most plausible.
**Conditional/ Unclassified**	A clinical event, including laboratory test abnormality, reported as an adverse reaction, about which more data is essential for a proper assessment or the additional data are under examination.	
**Unassessable / Unclassifiable**	A report suggesting an adverse reaction which cannot be judged because information is insufficient or contradictory, and which cannot be supplemented or verified.	
